# Knowledge, attitude, acceptance, and practice of COVID-19 vaccination and associated factors complemented with constructs of health belief model among the general public in South Gondar, Ethiopia: A community-based study

**DOI:** 10.3389/fpubh.2022.914121

**Published:** 2022-11-17

**Authors:** Hiwot Yisak, Birhanie Ambaw, Emaway Belay, Tsion Desalegn, Adanech Getie, Meswat Asrat, Asrate Guangul, Tigist Seid, Edgeit Abebe Zewde, Mengesha Assefa, Amien Ewunetei

**Affiliations:** ^1^Department of Public Health, College of Health Sciences, Debre Tabor University, Gondar, Ethiopia; ^2^Department of Midwifery, College of Health Sciences, Debre Tabor University, Gondar, Ethiopia; ^3^Department of Pharmacy, College of Health Sciences, Debre Tabor University, Gondar, Ethiopia; ^4^Department of Biomedical Sciences, College of Health Sciences, Debre Tabor University, Gondar, Ethiopia

**Keywords:** COVID-19 vaccination, acceptance, knowledge, attitude, Health Belief Model

## Abstract

**Introduction:**

Understanding the epidemiological dynamics of disease control, as well as the effectiveness, compliance, and success of the vaccination program requires an understanding of the local population's knowledge, attitude, and practice regarding the Corona Virus Disease of 2019 (COVID-19) vaccine. Thus, the objective of this study was to assess knowledge, attitude, and practice toward COVID-19 vaccination and associated factors among the general public.

**Methods:**

A cross-sectional study was conducted in the South Gondar Zone, among residents above the age of 18 years. The study used objective measures and the constructs of the Health Belief Model. Binary logistic regression was used and the result of the final model was presented in terms of adjusted odds ratio (AOR) and 95% confidence intervals (CI), and statistical significance was taken and considered at a *P*-value < 0.05.

**Results:**

The study was conducted on 1,111 study participants. The mean age is 30.83 ± 7.106. About 575 (51.8%) of the respondents have good knowledge about the COVID-19 vaccination and 43.4% have a positive attitude toward COVID-19 vaccination. About 361 (32.5%) of the respondents were willing to take the vaccine if it is available and 113 (10.2%) of them were vaccinated. Participants with a positive attitude and good knowledge, those with a secondary level of education AOR = 5.70, 95% CI (2.60–12.60), those with a monthly income of >2,000 birr AOR = 6.30, 95% CI (2.50–15.60), those having a television (TV), and those who use Facebook AOR = 17.70, 95% CI (10.10–30.90) had a higher level of acceptance of COVID-19 vaccination. The Health Belief Model's constructs of perceived susceptibility AOR = 1.53, 95% CI (1.26–1.85), perceived benefit AOR = 1.49, 95% CI (1.28–1.75), and cues to action AOR = 0.54, 95% CI (0.45–0.65) were all linked to COVID-19 vaccine acceptability.

**Conclusion:**

The level of acceptance of COVID-19 vaccination is much lower. Having a positive attitude score and good knowledge score, level of education, monthly income, presence of TV, the use of Facebook, and knowing the means of transmission of COVID-19 increase the level of acceptance of COVID-19 vaccination.

## Introduction

A contagious respiratory infectious disease caused by severe acute respiratory syndrome coronavirus 2 (SARS-CoV-2) has wreaked havoc on the world. On 12 March 2020, the World Health Organization (WHO) declares it a pandemic. The virus has infected nearly every country on the planet, and the number of deaths is rapidly rising (1). As of 3 February 2022, more than 385 million cases have been identified and almost 6 million deaths reported, both numbers are likely a gross underestimate of the extent of the SARS-CoV-2 spread (2). Incorrect attitudes and practices regarding preventive measures may also increase the risk of infection directly (3). Working with infected patients in overcrowded hospitals and resource-limited settings, including a lack of isolation rooms and inadequate awareness of infection prevention practices, healthcare workers and family members are at an increased risk of stress and mental health problems (4).

Vaccines represent the most effective prophylactic strategy in this era for controlling the spread of infectious diseases and have increased human life expectancy (5). Even though unspecified interventions, such as social distancing and quarantine can slow the spread of the virus, the COVID-19 epidemic will not end until herd immunity is well established through infection or vaccination, and vaccines are the most important public health measure and most effective strategy to protect the population from COVID-19 because SARS-CoV-2 is a highly contagious virus that affects populations widely and globally (6, 7).

The WHO Strategic Advisory Group of Experts (SAGE) defined vaccine hesitancy as a “delay in accepting or refusing vaccination despite the availability of vaccination services”. Three factors influence vaccine acceptability, such as confidence, convenience, and complacency. Confidence is defined as trust in the vaccine's effectiveness and safety, trust in the delivery system as the healthcare system, and trust in policymakers. Furthermore, vaccination convenience refers to the ease with which the vaccine can be obtained, which includes physical availability, affordability, and accessibility. Vaccine complacency is linked to a low noticed risk of vaccine-preventable disease and, as a result, more negative attitudes against vaccines (8).

There have been many COVID-19 cases on the African continent, and many people have died as a result. Fourteen African countries, including Ethiopia, are experiencing a new wave of the epidemic, which is on the rise (9).

World Health Organization (WHO) and CDC have initiated a multidisciplinary approach to combat COVID-19. Preventive measures aim to minimize person-to-person exposure, such as social distancing, self-isolation during symptoms, hand washing, and disinfection of surfaces. The World Health Organization, the Centers for Disease Control and Prevention, and the Ethiopian Ministry of Health, all recommended COVID-19 vaccination as the best way to control the pandemic in Ethiopia. Currently, it becomes a hot topic for most frontline healthcare providers including other personal protective equipment (10–12).

In the world including Ethiopia high-risk groups for initial vaccine supply were considered due to an insufficient supply of COVID-19 vaccines. Healthcare workers, the elderly, especially those with chronic comorbid conditions, and people working in essential services are among the high-risk groups (11, 13). Africa receives <2% of the 690 million COVID-19 vaccine doses administered worldwide. Vaccines have been delivered to 45 African countries, 43 of which have begun vaccination, and nearly 13 million of the 31.6 million doses delivered so far have been administered (9).

No vaccine has a safety threat and the efficacies are 95% for COVID-19 mRNA vaccine BNT162b2 (Pfizer), 94.1% for the mRNA-1273 vaccine (Moderna), 70.4% for ChAdOx1 nCoV-19 vaccine/AZD1222 (AstraZeneca) vaccine, and 78% for Sinovac, respectively (14).

Vaccination, despite being one of the most effective public health initiatives, is increasingly seen as unsafe and unnecessary by a growing number of people. The lack of trust in vaccines is now seen as a threat to vaccination programs' success. Vaccine apprehension is thought to be the cause of lower vaccine coverage and a higher risk of vaccine-preventable disease outbreaks (15). However, many groups and individuals have recently started spreading anti-vaccination rumors. Coronavirus disease 2019 (COVID-19) vaccine development and delivery is an ongoing process (16). In low- and middle-income countries, vaccination is at risk of being delayed for a variety of reasons including lack of public trust, lack of resources, and vaccine shortages (17). Even among healthcare workers, there is a lack of acceptance; studies show that not all healthcare workers are willing to accept COVID-19 vaccines if they are available in their country (18, 19). In the Democratic Republic of Congo, for example, a study found that about 28% of health workers would be willing to receive the COVID-19 vaccine if it were available (20). The slow vaccination coverage rate is partly caused by problems with vaccine distribution, such as a lack of health infrastructure and personnel. However, there are also concerns that vaccine hesitancy and disbelief may play a role (21). Misconceptions also contribute to widespread myths and fears associated with infectious disease clinical trials. However, such fears are frequently linked in various ways to a legacy of mistrust stemming from past medical malpractice and unethical experimentation, which has resulted in major international litigation in some cases (22, 23). Understanding the local population's knowledge, attitudes, and practices (KAP) or acceptance of the COVID-19 vaccine is vital. For example, a study conducted in China which entitled with a behavioral intention to get a booster dose of COVID-19 vaccine among Chinese factory workers demonstrated that perceptions related to a booster dose as well as interpersonal level factors, such as information exposure on social media, thoughtful consideration of the veracity of the information, and satisfaction with vaccine-related promotional materials were determinants of behavioral intention (24).

The epidemiological dynamics of disease control, as well as the vaccination program's effectiveness, adherence, and success depends on knowledge attitude and practices toward COVID-19 vaccination. Thus, this study aimed to assess the public knowledge, attitude, and acceptance/practice of COVID-19 vaccination and also the predictors in urban districts of the South Gondar Zone complementing the Health Belief Model (HBM). HBM hypothesizes that health-related behavior depends on the combination of several factors, namely, perceived susceptibility, perceived severity, perceived benefits, perceived barriers, cues to action, and self-efficacy.

## Materials and methods

### Study area

The research was carried out in the eight special districts of the South Gondar Zone (Debre Tabor, Addis Zemen, Woreta, Nefas mewcha, Mekane-eyesus, Hamusit wegeda, and Ebinat). In the zone, there are 96 health centers, 7 primary hospitals, and 1 general hospital. The South Gondar Zone has a total population of 2,239,077 people, according to the 2011 CSA.

### Study design and period

A cross-sectional study was conducted from 1 October 2021 to 1 December 2021 complementing approaches with the constructs of the Health Belief Model.

### Source and study population

Residents of the South Gondar Zone.

### Inclusion criteria and exclusion criteria

Adults above the age of 18 years.

People resided for at least 6 months in the South Gondar Zone special.

Those who are not able to communicate were excluded.

### Sample size determination

The representative sample size for the general public is determined by using Epi Info 7 by taking a proportion of 50%, a margin of error to be 3%, and a confidence level of 95%. The final sample size after adding 10% non-response is 1,175.

### Sampling methods and procedures

First, the census was conducted in all the kebeles to estimate the total population. Then, the total sample size was distributed to all kebeles proportionally to their population size, and the participants of the study were selected using a systematic random sampling method after determining the sample interval from the total population in each kebele. We determined the starting point using household numbers and to identify the initial we used the lottery method. Then, for those with two or more eligible individuals in the household, the lottery method was used to select one individual.

### Variables

#### Dependent variables

Knowledge, attitude, and acceptance/practice of COVID-19 vaccination.

#### Independent variables

Sociodemographic/economic factors: Age, Religion, Occupational status, Educational status, residence, marital status, distance to the nearby health facility, income, Family size, and Presence of Television and Radio, Personal and behavioral factors: Presence of comorbidities (DM, hypertension, cancer, HIV/AIDS, asthma), being infected with COVID-19, and Use of social media (Facebook, Telegram).

### Operational definitions

The level of knowledge was assessed using structured questionnaires and it was divided into good knowledge and poor knowledge. Respondents with scores greater than or equal to the mean value of the sum of knowledge assessment questions were considered to have good knowledge, while those with scores lower than the mean value of the sum of knowledge assessment questions were considered to have poor knowledge. The attitude was assessed using structured questionnaires and it was divided into good (favorable) attitudes and bad (unfavorable) attitudes.

Respondents with a positive (favorable) attitude were thought to have a score greater than or equal to the mean value of the sum of attitude-related questions, while those with a negative (unfavorable) attitude were thought to have a score less than the mean value of the sum of attitude assessment questions.

Level of acceptance was assessed by question (Did you have an intention to accept COVID-19 vaccine if it is available in the future; respondents who respond “yes” and “No”).

The practice was assessed by health professionals (by the question, did you take the vaccine? Yes or no).

For the constructs of HBM, five items response will be prepared for each construct strongly agree (scores 5 points) to strongly disagree (scores 1 point), perceived the susceptibility of COVID-19 consisted of 3 items scored from 3 to 15, the seriousness of COVID-19 contains 6 items scored from 6 to 30, benefits consisted of 5 items scored from 5 to 25, barriers for practicing prevention measures consisted of 14 items scored from 14 to 70, self-efficacy consisted of 7 items scored from 7 to 35, and cues to action consisted of 6 items scored from 6 to 30. For all constructs of HBM, the higher scores indicated having a high perception toward practicing COVID-19 vaccination measures, but a higher score for barriers indicated a high barrier to practicing COVID-19 vaccination measures. For perceived net benefit, we used the sum score of perceived benefit minus perceived barriers. The practice of COVID-19 vaccination methods will be assessed by “Yes” or “No” type questions.

### Data collection tools and procedures

The questionnaire was developed after reviewing different works of literature or studies of COVID-19 conducted in Ethiopia and other studies conducted in Africa and other continents. For the socioeconomic and demographic parts, we have adopted DHS guidelines. The questionnaire was first prepared in the English version and translated to the local language (Amharic); then, it was retranslated again to English by language experts to ensure consistency. The data collectors were eight B.Sc nurses and five public health officers on each site. Two supervisors (public health experts) were assigned for supervising the data collectors. World Health Organization (WHO) recommendations for the prevention of COVID-19 by social distancing, use of personal protective equipment, and alcohol-based hand sanitizer were used during data collection.

### Assurance of data quality

The questionnaire was created after an extensive search and review of relevant studies on the topic to ensure data quality. The questionnaire was translated into the local language (Amharic) to facilitate communication. The supervisor and data collectors received 2 days of training on how to collect data, study overviews, questionnaires, and other data collection activities. In addition, the structured questionnaire was pretested on 5% of the total sample size outside the study area. After the pretest, difficult questions were revised and modification was carried out. The pretested data were not included in the main data. Throughout the data collection period, strict daily supervision of the data collection process was maintained. Supervisors visited study sites daily and received completed questionnaires after double checking their accuracy. Incomplete questionnaires were returned to the data collector the questionnaire to be completed again.

### Data processing and analysis

Before the data were processed, it was double checked for accuracy and consistency. The information was then coded and entered into Epi Info version 7.2 before being exported to the software SPSS version 23 for analysis. The analyses were confirmed using descriptive interpretation for the study participants' demographic and socioeconomic characteristics, as well as frequencies and other summary statistics. The association of each covariate with the outcome variable was measured using binary logistic regression. To control for potential confounders, factors that were associated with the outcome variable at a 20% significance level were included in the multivariable logistic regression analysis. The final model's results were presented as adjusted odds ratio (AOR) and 95% confidence intervals (CI). If the *p*-value is <0.05, then the data were considered statistically significant.

### Ethics statement

Debre Tabor University gave their ethical approval. A formal letter was sent to all of the districts' concerned bodies, requesting their cooperation in facilitating the study. The purpose, benefits, and risks of the study were explained to the interviewers to obtain informed written consent from the study participants before data collection. The study participants were informed that the information they provided would be kept confidential and would not be used for any purpose other than the study. The information they provided did not include their names, the names of their children, or any other identifiers that could be used to identify them.

## Results

### Socioeconomic and sociodemographic characteristics

The study was conducted on 1,111 study participants with a response rate of 94.5%. The mean age was 30.83 ± 7.106. Approximately 707 (63.6%) of the study participants belong to the age group of 25–35 years. Of the total respondents, men account for 577 (51.9%). The majority of the respondents 982 (88.4%) have a family size of <5 and married participants account for 853 (76.8) of the total study participants. Of the total respondents, about 730 (65.7%) were civil servants and 316 (28.4%) of the respondents earn a monthly income of <2,000 birr. Regarding media use, 913 (82.2%) of the respondents have a TV in their house, 853 (76.8%) of the respondents use Facebook, and 193 (17.4%) of the respondents did not use any social media (refer to Table 1).

**Table 1:** Socioeconomic and sociodemographic characteristics of study participants.

**Variables**		**Frequency**	**Percent**
Age	<25	210	18.9
	25–35	707	63.6
	35–45	156	14.0
	45–55	29	2.6
	>55	9	0.8
Sex	Male	577	51.9
	Female	534	48.1
Family size	<5	982	88.4
	≥5	129	11.6
Religion	Orthodox	1,061	95.5
	Muslim	50	4.5
Marital status	Single	248	22.3
	Married	853	76.8
	Divorced	10	0.9
What is your level of	Unable to read and write	38	3.4
education?	Able to read and write	9	0.8
	The primary level of education	108	9.7
	Secondary level of education	112	10.1
	College and above	844	76.0
What is your	Self-employed	134	12.1
occupational status?	Civil servant	730	65.7
	Student	94	8.5
	Farmer	34	3.1
	Other	119	10.7
Monthly income	<2,000	316	28.4
	2,000–5,000	214	19.3
	>5,000	581	52.3
Distance from the	<1,000 m	735	66.2
health facility	1,000–5,000 m	268	24.1
	>5,000 m	108	9.7
Presence of TV	Yes	913	82.2
	NO	198	17.8
Presence of radio?	Yes	381	34.3
	NO	730	65.7
Use of Facebook	Yes	853	76.8
	No	258	23.2
Use of telegram	Yes	616	55.4
	NO	495	44.6
Use of other social	Yes	121	10.9
media	NO	990	89.1
No use social media	Yes	193	17.4
use	NO	918	82.6

### Health status of study respondents and COVID-19-related information

Of the total respondents, about 120 (10.8%) of the respondents had chronic diseases. Sixty-one (5.5%) of the study participants personally believe that they belong to a vulnerable group with chronic non-communicable diseases. Fifty-five (5.0%) believed that they are living with someone belonging to a vulnerable group of COVID-19. Eighty-two (7.4%) of the respondents reported that they have asthma. Approximately 959 (86.3%) of the respondents reported fever as a sign of COVID-19, 798 (71.8%) of the respondents reported cough as a symptom of COVID-19, and 53 (4.8%) of the respondents do not know any signs and symptoms of COVID-19. Sixty-four (5.8%) of the respondents believe that COVID-19 has no signs and symptoms. Approximately 861 (77.5%) of the respondents reported inhalation of respiratory droplets of an infected person as a means of transmission of COVID-19. Of the total respondents, 884 (79.6%) responded that wearing a facemask can prevent the transmission of COVID-19, and 140 (12.6%) of the respondents believe that consuming herbs can prevent the transmission of COVID-19 (Table 2).

**Table 2:** Health status of study respondents and COVID-19-related information.

			**Frequency**	**Percentage**
Chronic diseases	Do you have any chronic diseases?	Yes	120	10.8
		NO	991	89.2
	Do you personally belong to a vulnerable group	Yes	61	5.5
	with chronic non-communicable disease?	NO	1,050	94.5
	Are you Living with someone belonging to a	Yes	55	5.0
	vulnerable group	No	1,056	95.0
		Total	1,111	100.0
Do you have these illnesses	Kidney Failure	Yes	9	0.8
		No	1,102	99.2
	Heart diseases	Yes	5	0.5
		No	1,106	99.5
	Anemia	Yes	47	4.2
		No	1,064	95.8
	Hypertension	Yes	27	2.4
		No	1,084	97.6
	Asthma	Yes	82	7.4
		No	1,029	92.6
	Hepatic diseases	Yes	24	2.2
		No	1,087	97.9
Signs and symptoms of COVID-19	Fever	Yes	959	86.3
		No	152	13.7
	Chills	Yes	365	32.9
		No	746	67.1
	Diarrhea	Yes	265	23.9
		No	846	76.1
	Cough	Yes	798	71.8
		No	313	28.2
	Otitis media	Yes	130	11.7
		No	981	88.3
	Loss of smell and taste senses	Yes	437	39.3
		No	674	60.7
	No symptoms	Yes	64	5.8
		No	1,047	94.2
	Don't know	Yes	53	4.8
		No	1,058	95.2
Means of transmission of COVID-19	Drinking unclean water	Yes	70	6.3
		No	1,041	93.7
	Eating unclean food	Yes	37	3.3
		No	1,074	96.7
	Inhalation of respiratory droplets of an	Yes	861	77.5
	infected person	No	250	22.5
		Total	1,111	100.0
	Eating or touching wild animals	Yes	178	16.0
		No	933	84.0
	Other	Yes	83	7.5
		No	1,028	92.5
Means of prevention of COVID 19	Wearing face masks	Yes	884	79.6
		No	227	20.4
	Washing hands with regular soap	Yes	848	76.3
		No	263	23.7
	Using detergents	Yes	458	41.2
		No	653	58.8
	Social distancing	Yes	779	70.1
		No	332	29.9
	Avoid touching face/mouth/nose/eyes	Yes	673	60.6
		No	438	39.4
	Consume vitamin C	Yes	261	23.5
		No	850	76.5
	Consume zinc	Yes	132	11.9
		No	979	88.1
	Avoid eating meat	Yes	110	9.9
		No	1,001	90.1
	Consume herbs	Yes	140	12.6
		No	971	87.4

### COVID-19 vaccine-related knowledge and attitude

Approximately 863 (77.7%), 95% CI (29.8–35.2) of the study participants heard about COVID-19 vaccines. The results of the current study demonstrated that about 575 (51.8%) of the respondents had good knowledge about the COVID-19 vaccination. Regarding the attitude of the respondents, 43.4%, 95% CI (40.5–46.3) had a positive attitude toward COVID-19 vaccination (Table 3).

**Table 3:** Knowledge and attitude of the respondents toward COVID-19 vaccinations.

**Knowledge and attitude**		**Frequency**	**Percent**
**toward COVID-19 vaccination**			
Knowledge about	Good knowledge	575	51.8
	Poor knowledge	536	48.2
Attitude COVID-19	Positive attitude	482	43.4
vaccination	Poor attitude	629	56.6

### Constructs of Health Belief Model on COVID-19 vaccination

**Figure 1:**
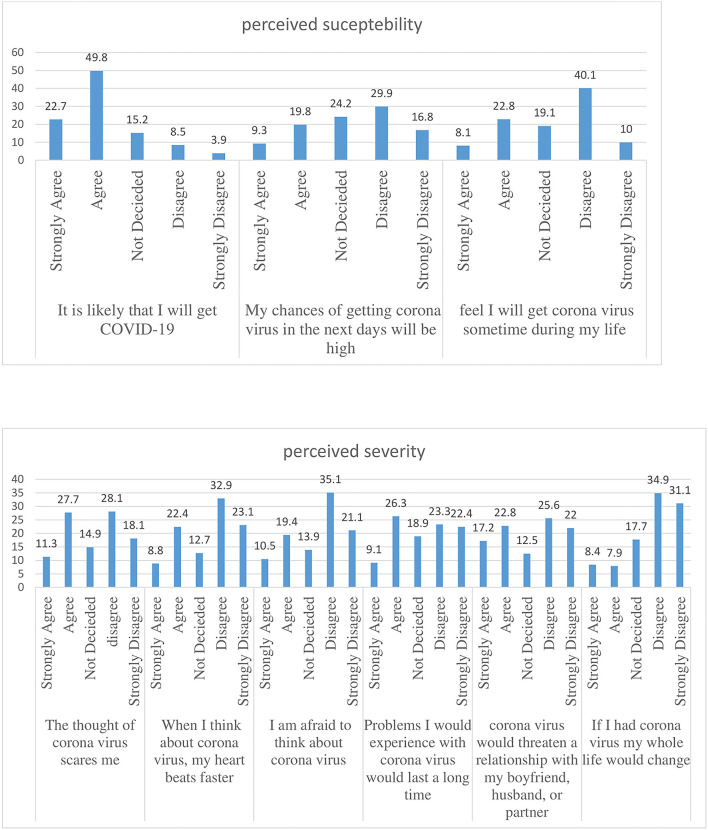
Percentage of perceived susceptibility and severity attitude toward COVID-19 of the study participants.

**Figure 2:**
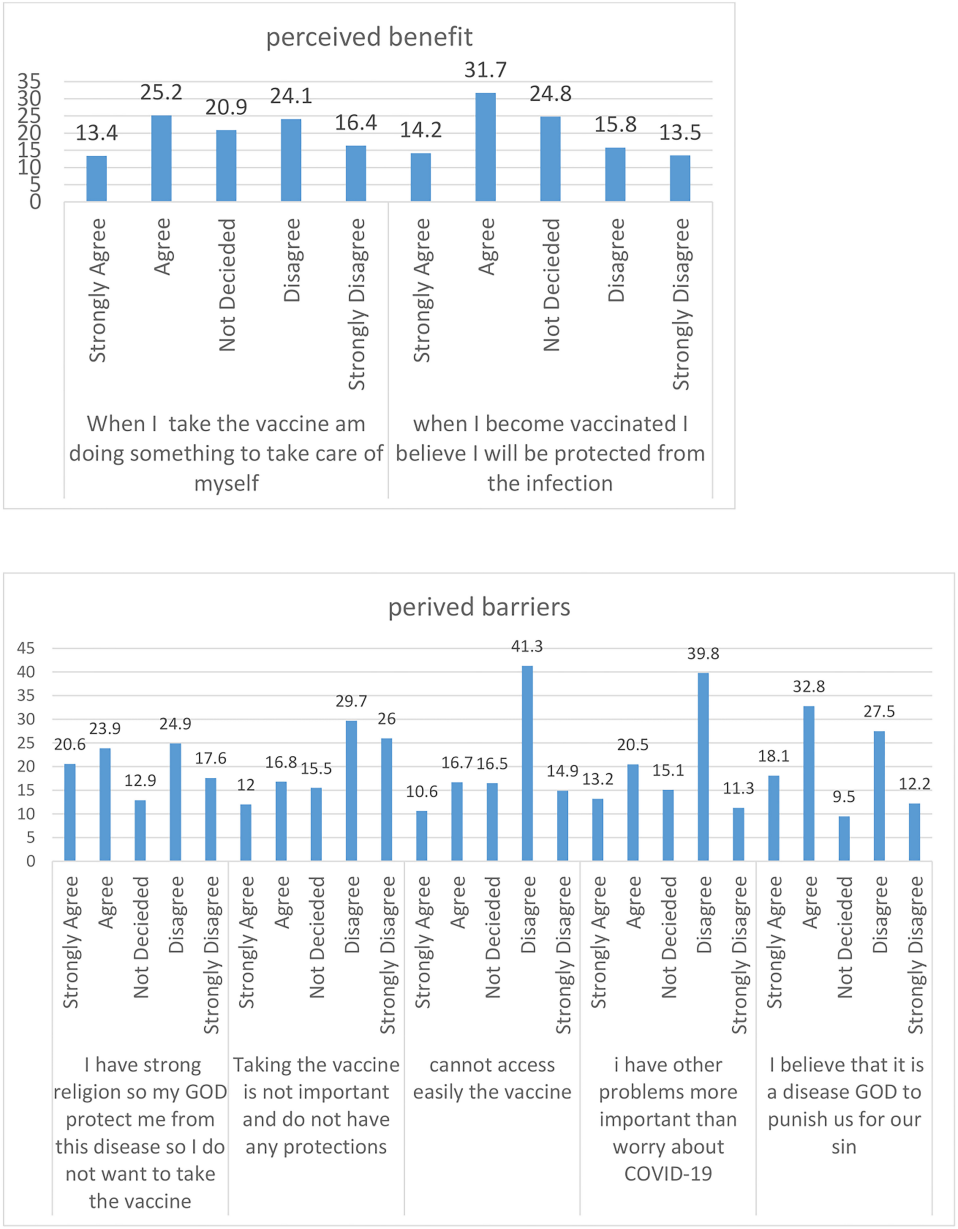
Percentage of perceived barriers and benefits attitude toward COVID-19 of the study participants.

**Figure 3:**
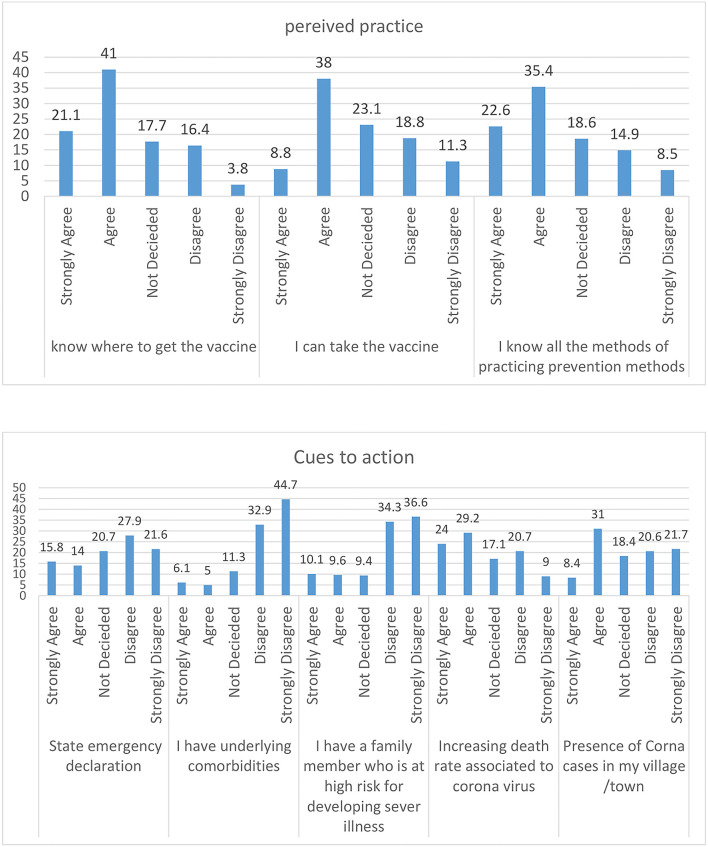
Percentage of practice and cues to action attitude toward COVID-19 of the study participants.

From the components of the Health Belief Model, almost half of the respondents, 553 (49.8%) agreed that it is likely that they will get COVID-19. Approximately 332 (29.9%) of the respondents disagreed that their chances of getting the coronavirus in the next few days will be high. Of the total respondents, 308 (27%) agreed that the thought of coronavirus scares them. Approximately 280 (25.2%) of the respondents agreed that when they take the vaccine they are doing something to take care of themselves. One hundred and two (9.2%) of the respondents strongly agreed that the vaccine is embarrassing to them and 239 (21.5%) agreed that the vaccine is not important. Four hundred and fifty-six (41.0%) agreed on where to get the vaccine. Approximately 289 (26%) of the respondents strongly disagreed that taking the vaccine is not important and does not have any protection. Approximately 364 (32.8%) of the study participants agreed that they believe that it is a disease of GOD to punish us for our sins. Approximately 393 (35.4%) of the participants agreed that they know all the methods of practicing prevention methods and 442 (39.8%) of the participants disagreed that they have other problems more important than worrying about COVID-19. Approximately 324 (29%) of the participants agreed that there is an increasing death rate associated with coronavirus (Figures 1–3).

### Acceptance and practice of the COVID-19 vaccine

Approximately 361 (32.5%) of the respondents were willing to take the vaccine if it is available and 113 (10.2%) of the respondents took the vaccination (vaccinated; Table 4).

**Table 4:** The acceptance and practice of COVID-19 vaccination among study participants.

**Practice and acceptability**		**Frequency**	**Percent**
**of the vaccine**			
Acceptability of the	Yes	361	32.5
vaccine	No	750	67.5
	Total	1,111	100.0
Took the vaccine	Yes	113	10.2
	No	998	89.8
	Total	1,111	100.0

### Factors associated with the acceptability of COVID-19 vaccinations

In the bivariate analysis, sex of the respondents, educational status, occupational status, availability of TV in the house, the use of Facebook, not knowing the means of transmission of COVID-19, consumption of vitamin C, zinc, and herbs, attitude score toward COVID-19 vaccination, and knowledge score about COVID-19 vaccination were associated with the acceptability of COVID-19 vaccination.

Finally, in the multivariable logistic regression, those with the educational status of secondary level of education were five times more likely to accept the vaccination as compared with those who were not able to read and write, AOR = 5.7,95% CI (2.6–12.6). Civil servants, AOR = 0.32, 95% CI (0.01–0.1) and farmers, AOR = 0.01, 95% CI (0.03–0.04) were less likely to accept the vaccine as compared with those who were self-employed. The monthly income of >2,000 birr increased the chance of the acceptability of COVID-19 six times, AOR = 6.3, 95% CI (2.5–15.6). The presence of TV decreased the chance of acceptability of the vaccine by 46%, AOR = 0.54, 95% CI (0.3–0.8). The use of Facebook increases the level of acceptability of COVID-19 vaccination by seventeen times with AOR = 17.7, 95% CI (10.1–30.9). Participants having a positive attitude score and good knowledge score are more likely to have good acceptance of the vaccine. Those participants who thought that consumption of vitamin C can prevent COVID-19 infection are less likely to have good acceptance of COVID-19 vaccination with AOR = 0.386, 95% CI (0.25–0.57). Knowing the means of transmission of COVID-19 increases the acceptability of COVID-19 vaccination by nineteen times, AOR = 19.2, 95% CI (4.8–26.1). From the constructs of the Health Belief Model, perceived susceptibility, AOR = 1.531, 95% CI (1.26–1.85), perceived benefit, AOR = 1.498, 95% CI (1.284–1.747), and cues to action, AOR = 0.54, 95% CI (0.446–0.653) were associated with the acceptability of COVID-19 vaccine (refer to Table 5).

**Table 5:** Factors associated with the acceptability of COVID-19 vaccination.

**Variables**		***P***	**AOR**	**95% C.I.for AOR**	
				**Lower**	**Upper**
Sex	Male	0.29	0.79	0.52	1.22
	Female	1	1	1	1
Educational status	Unable to read and write	1	1	1	1
	Able to read and write	0.01	0.11	0.02	0.51
	The primary level of education	0.99	182	0.00	239
	Secondary level of education	0.00	5.71	2.59	12.59
	College and above	0.41	0.78	0.43	1.40
Occupational status	Self-employed	1	1	1	1
	Civil servant	0.00	0.03	0.01	0.10
	Student	0.44	0.63	0.19	2.03
	Farmer	0.00	0.01	0.00	0.04
	Other	0.42	2.08	0.35	12.34
Monthly income	<2,000	0.00	1	1	1
	2,000–5,000	0.00	6.30	2.50	15.60
	>5,000	0.01	1.90	1.20	3.30
Presence of TV	Yes	0.01	0.54	0.35	0.84
	No	1	1	1	1
Use of Facebook	Yes	0.00	17.70	10.11	30.99
	No	1	1	1	1
Knowing the means of transmission of COVID-19	Yes	0.00	19.20	4.84	76.16
	No	1	1	1	1
Consumption of vitamin C for prevention of COVID 19	Yes	0.00	0.38	0.25	0.57
	No				
Knowledge score	Good	0.01	1.73	1.18	2.53
	Poor	1	1	1	1
Attitude score	Good	0.00	7.73	5.17	11.55
	Poor	1	1	1	1
Constructs of health belief model	SUSS	0.00	1.53	1.26	1.859
	SERR	0.00	3.31	2.72	4.01
	BEN	0.00	1.450	1.28	1.75
	BARR	0.01	0.78	0.64	0.95
	PRA	0.51	1.07	0.88	1.31
	CUESS	0.00	0.54	0.45	0.65

### Factors associated with the practice of COVID-19 vaccination

Sex of the respondents, family size, educational status, presence of chronic disease, presence of anemia, knowing the means of transmission of COVID-19, knowledge score, attitude score of COVID-19 vaccination, and use of Facebook and Telegram were associated with the practice (intake) of COVID-19 vaccination in the bi-variable logistic regression. In the multivariable logistic regression, males were less likely to take the vaccination with AOR = 0.14, 95% CI (0.07–0.28). Participants who have an educational status of college and above were less likely to take the vaccination with AOR = 0.37, 95% CI (0.17–0.83) and not knowing the means of transmission of COVID-19 decreases the chance of vaccination by 96%, AOR = 0.04, 95% CI (0.015–0.13). Those participants having positive attitude scores were more likely to take the vaccination with AOR = 28, 95% CI (9.03–87.3). Those participants using Telegram were less likely to take COVID-19 vaccination, AOR = 0.18, 95% CI (0.08–0.39) (Table 6).

**Table 6:** Factors associated with the practice of COVID-19 vaccination.

**Variables**		***p***	**AOR**	**95% C.I.for AOR**	
				**Lower**	**Upper**
Sex	Male	0.00	0.14	0.07	0.28
	Females	1	1	1	1
Family size	<5	0.08	0.50	0.23	1.10
	>5	1	1	1	1
Educational status	Unable to read and write	1	1	1	1
	Able to read and write	0.01	0.19	0.05	0.74
	Primary level of education	0.98	5.30	0.54	25.70
	Secondary level of education	0.99	11.1	0.01	39.80
	College and above	0.01	0.37	0.17	0.83
Presence of chronic disease	Yes	0.99	185	0.8	190
	No	1	1	1	1
Anemia	Yes	1	1	1	1
	No	0.00	0.04	0.01	0.13
Knowing means of transmission of COVID-19	Yes	1	1	1	1
	No	0.00	0.04	0.02	0.14
Knowledge score	Good	0.00	0.09	0.03	0.24
	Poor				
Attitude score	Good	0.00	28.08	9.03	87.30
	Poor				
Facebook use	Yes	0.97	1.01	0.43	2.36
	No				
Telegram use	Yes	0.00	0.18	0.08	0.39
	No				

## Discussion

The knowledge, attitude, acceptance, and practice of the COVID-19 vaccination, as well as associated factors, were assessed in the South Gondar Zone of Ethiopia. In the current study, 51.8% of study participants had good knowledge about the vaccine which is lower compared with studies from Northeast Ethiopia (62%) (25), Saudi Arabia (76%) (26), and China (91.3%) (27). The differences may be due to the different sociodemographic backgrounds and because our study is conducted in the general population while the study in Northeast Ethiopia was conducted among healthcare workers. Healthcare workers are on the front lines of the pandemic and have better access to information toward the vaccine.

In the current study, about 863 (77.7%) of the study participants heard about COVID-19 vaccines. Approximately 361 (32.5%) of the respondents are willing to take the vaccine if it is available and 113 (10.2%) of the respondents took vaccination (vaccinated). Even though the results of the current study demonstrated that about 575 (51.8%) of the respondents have good knowledge about COVID-19 vaccination, 43.4% have a positive attitude toward COVID-19 vaccination, the level of acceptance of COVID-19 vaccination in the current study is much less than most of the studies. For example, a narrative review on vaccine hesitancy in the era of COVID-19 which included 15 studies showed that the percentage of COVID-19 vaccine acceptance was up to 86.1% for students or 77.6% for the general population (28). In another study on COVID-19 vaccination acceptance and its associated factors among the Middle Eastern population 6.8% of the participants are not willing to take the vaccine and 26.4% were not sure of it (29).

The pooled COVID-19 vaccine acceptance rate was 73.31%, according to a systematic review and meta-analysis of acceptability and its predictors (95% CI: 70.52, 76.01). Gender, educational level, history of influenza vaccination, and government trust were all strong predictors of COVID-19 vaccination willingness (30). Another study which was conducted among the adult general population of Greece indicates that a significant proportion of individuals are willing to receive the COVID-19 vaccine, 57.7% are going to get vaccinated for COVID-19 when the vaccine is available, while 26.0% are unwilling to receive the COVID-19 vaccine and 16.3% are unsure (31).

According to a study conducted in the West Indian community, nearly 79% of study participants were willing to take the COVID-19 vaccine when it became available, while only 2% were against it. Others were undecided about their response (32).

According to a study conducted in India, the majority of people were willing to get vaccinated as soon as the opportunity arose (83.6%), willing to pay for the vaccine (75.43%), and willing to recommend it to their family and friends (82.77%) (33).

According to a study conducted in Hong Kong, 42.2% of study participants expressed their acceptance of the COVID-19 vaccine, while 17.4% expressed opposition, and 40.4% indicated that they were unsure (34). According to a survey of Bangladeshi adults, 74.6% said they would be willing to get vaccinated against COVID-19 if a safe and effective vaccine was available without charge, while 8.5% said they would be hesitant (35).

According to a study conducted among Saudi adults, about 48% of them were willing to receive the COVID-19 vaccine. If they lived in the southern region, had previously received seasonal influenza vaccination, believed in mandatory COVID-19 vaccination, or expressed high levels of concern about contracting COVID-19, participants were more likely to receive a vaccination. Participants were less likely to have the intention to be vaccinated if they had a history of vaccine refusal (36). The reason for less acceptance might be the late introduction and dissemination of the vaccine in the country due to different political conditions of the country; there was war in different parts and some parts of the study area which may shift or change people's thoughts toward COVID-19.

The results of the current study demonstrated that about 575 (51.8%) of the respondents have good knowledge about COVID-19 vaccination. Regarding the attitude of the respondents, 43.4% have a positive attitude toward COVID-19 vaccination. Similarly, a study which was conducted in India indicates that people with allergies (57.89%) and immune-compromised patients (62.98%), pregnant and lactating women (41.89%), and patients with chronic illness (34.78%) know vaccine eligibility. Overall, people have a positive attitude toward vaccines (33).

Thus, to fill the knowledge, attitude, and perception gap, health information dissemination and health education about COVID-19 vaccination should be given to the community.

The finding of the current study indicated that those with the educational status of secondary level of education and those using Facebook were more likely to have higher vaccine acceptability, similar findings have been from other studies (37, 38). A higher level of education increases access to information and concern about health which increases vaccine acceptability (39). Having the occupation of civil servants and farmers and the presence of TV decrease the chance of acceptability of the COVID-19 vaccine. Similarly, a study which was conducted among Bangladeshi adults indicates that COVID-19 vaccine refusal was found to be significantly higher in the elderly, rural, semi-urban, and slum communities, farmers, day laborers, homemakers, low-educated communities, and those who had little faith in the country's health service (35). Overall, according to a systematic review and meta-analysis of COVID-19 vaccine acceptability and predictors, the pooled COVID-19 vaccine acceptance rate was 73.31% (95% CI: 70.52, 76.01). Gender, educational level, influenza vaccination history, and administration trust were all strongly predictive of willingness to get vaccinated against COVID-19 (30).

In the current study being male, having an educational status of college and above, not knowing the means of transmission of COVID-19, and using Telegram were associated with not being vaccinated with COVID-19 vaccination. Those participants having positive attitude scores were more likely to take the vaccination. On the other hand, a study on COVID-19 vaccination hesitancy or acceptance and associated factors in Punjab Pakistan showed that sociodemographic factors including male, middle or higher level of education, and high access to mass media were significantly associated with COVID-19 vaccination uptake (40). Being younger (18–30 vs. 41–50 years), having a lower education level, being employed, and belonging to priority groups for vaccination were all linked to increased odds of acceptance in a cross-sectional study in China (41).

The proportion of participants with a high likelihood of receiving a COVID-19 vaccine was 62.1%, according to a study conducted in Japan. The result showed that from the study participants women, adults aged 20–49 years, and those with a low-income level had lower vaccine acceptance. Vaccine acceptance was linked to several psychological factors, including the perceived effectiveness of the COVID-19 vaccine and the willingness to protect others by getting vaccinated. The acceptance of the COVID-19 vaccine may also be influenced by the vaccine's perceived effectiveness and willingness to protect others (42). Another study found that men (OR = 4.35, 95% CI: 1.58–11.93) and educated respondents (OR = 3.54, 95% CI: 1.44–8.67) were more likely to say they wanted to get the COVID-19 vaccine (43).

In the current study, those participants having a positive attitude score and good knowledge score are more likely to have good acceptance of the vaccine. Those participants who thought that consumption of vitamin C can prevent COVID-19 infection are less likely to have good acceptance of COVID-19 vaccination; those participants knowing the means of transmission of COVID-19 increases the acceptability of COVID-19 vaccination.

There are several theories and models that support the practice of health promotion and disease prevention. When identifying a theory or model to guide health promotion or disease prevention programs, it is important to consider a range of factors, such as the specific health problem being addressed, the population(s) being served, and the contexts within which the program is being implemented. Selected theories and models that are used for health promotion and disease prevention programs include ecological models, models, stages, social cognitive theory, and theory of reasoned action/planned behavior (44). On the other hand, there is a study that investigated the psychological drivers of vaccination intention using the 5C model as a mediator. The model includes five antecedents of vaccination: confidence, complacency, constraints, calculation, and collective responsibility (45). The current study used the Health Belief Model (HBM).

Perceived susceptibility is one of the constructs of the Health Belief Model. The acceptability of the COVID-19 vaccine was linked to perceived benefits and cues to action. According to a study conducted in Hong Kong, perceived severity, perceived vaccine benefits, cues to action, self-reported health outcomes, trust in the healthcare system or vaccine manufacturers, and government recommendations were positive correlates of acceptance, whereas perceived access barriers and harm were negative correlates (34). Preventive measures, perceived benefit, perceived barriers, cues to action, subjective norm, support of vaccination in general, and having received a flu vaccine before were all found to be important factors in the acceptance of the COVID-19 vaccine among healthcare workers and the general population, according to a study using the Health Belief Model (46). Participants were more likely to be willing to get vaccinated if they reported higher levels of perceived benefits of the COVID-19 vaccine (OR = 4.49, 95% CI: 2.79–7.22), perceived severity of COVID-19 infection (OR = 2.36, 95% CI: 1.58–3.51), and cues to action (OR = 1.99, 95% CI: 1.38–2.87) in a study on predicting intention to receive COVID-19 vaccine among the general population using the Health Belief Model (HBM) (43).

### Strengths and limitations of the study

The strengths of this study are that the study was community-based in that it can represent the source population and the sample size is also large. On the other hand, the limitations of the study were the study design (cross-sectional), most of the data are self-reported data, and there might be social desirability bias and recall bias.

## Conclusion

The level of acceptance of COVID-19 vaccination is much lower. Having a positive attitude score and good knowledge score, level of education, monthly income, the presence of TV, and the use of Facebook. Knowing the means of transmission of COVID-19 increases the level of acceptability of COVID-19 vaccination. Perceived susceptibility and perceived benefits were positively associated with the acceptability of the COVID-19 vaccine. Being male, having an educational status of college and above, not knowing the means of transmission of COVID-19, and using Telegram were associated with not being vaccinated by COVID-19 vaccination. Those participants having positive attitude scores were more likely to take the vaccination. Thus, to increase the coverage/usage of COVID-19 vaccination, we recommend interventions scaling up knowledge and attitude and promoting the use of Facebook and TV.

## Data availability statement

The original contributions presented in the study are included in the article/supplementary material, further inquiries can be directed to the corresponding author.

## Ethics statement

The studies involving human participants were reviewed and approved by Debre Tabor University Research Ethics Review Committee. The patients/participants provided their written informed consent to participate in this study.

## Author contributions

All authors made significant contributions to the conception and design, data acquisition, data analysis and interpretation, contributed to the article's drafting and critical revision of important intellectual content, agreed to submit to the current journal, gave approval for the published version, and agreed to be responsible for all aspects of the study.

## Conflict of interest

The authors declare that the research was conducted in the absence of any commercial or financial relationships that could be construed as a potential conflict of interest.

## Publisher's note

All claims expressed in this article are solely those of the authors and do not necessarily represent those of their affiliated organizations, or those of the publisher, the editors and the reviewers. Any product that may be evaluated in this article, or claim that may be made by its manufacturer, is not guaranteed or endorsed by the publisher.

## Abbreviations

AIDS: acquired immunodeficiency syndrome
AOR: adjusted odds ratio
CDC: center for disease control
CI: confidence interval
COVID-19: Corona Virus Disease of 2019
CSA: central statistics agency
DM: diabetes mellitus
HCWs: health care workers
HIV: human immunodeficiency virus
KAP, knowledge, attitude: and practice
SAGE: strategic advisory group of experts
SARS-CoV-2: severe acute respiratory syndrome Coronavirus 2
WHO: World Health Organization.

## References

[1] Al-Tawfiq JA Leonardi R Fasoli G Rigamonti D. Prevalence and fatality rates of COVID-19: what are the reasons for the wide variations worldwide? Travel Med Infect Dis. (2020) 35:101711. 10.1016/j.tmaid.2020.101711 32360326 PMC7188640

[2] Alexandridi M Mazej J Palermo E Hiscott J. The coronavirus pandemic−2022: viruses, variants and vaccines. Cytokine Growth Factor Rev. (2022) 63:1–9. 10.1016/j.cytogfr.2022.02.002 35216872 PMC8839804

[3] Bai W Cai H Liu S Liu H Qi H Chen X. Attitudes toward COVID-19 vaccines in Chinese college students. Int J Biol Sci. (2021) 17:1469–75. 10.7150/ijbs.58835 33907510 PMC8071773

[4] Wu Z McGoogan JM. Characteristics of and important lessons from the coronavirus disease 2019 (COVID-19) outbreak in China: summary of a report of 72,314 cases from the Chinese center for disease control and prevention. JAMA. (2020) 323:1239–42. 10.1001/jama.2020.2648 32091533

[5] Sautto GA Kirchenbaum GA Diotti RA Criscuolo E Ferrara F. Next generation vaccines for infectious diseases. J Immunol Res. (2019) 2019:5890962. 10.1155/2019/5890962 31183388 PMC6515045

[6] Chan EY-Y Cheng CK-Y Tam GC-H Huang Z Lee PY. Willingness of future A/H7N9 influenza vaccine uptake: a cross-sectional study of Hong Kong community. Vaccine. (2015) 33:4737–40. 10.1016/j.vaccine.2015.07.046 26226564

[7] Wibawa T. COVID-19 vaccine research and development: ethical issues. Trop Med Int Health. (2021) 26:14–9. 10.1111/tmi.13503 33012020 PMC7675299

[8] MacDonald NE. Vaccine hesitancy: definition, scope and determinants. Vaccine. (2015) 33:4161–4. 10.1016/j.vaccine.2015.04.036 25896383

[9] Africa W. Less Than 2% of World's COVID-19 Vaccines Administered in Africa. Geneva: World Health Organization (2021).

[10] COVID C. Overview and Infection Prevention and Control Priorities in Non-US Healthcare Settings. Atlanta, GA: Centers for Disease Control and Prevention (2020).

[11] Africa W. Ethiopia Introduces COVID-19 Vaccine in a National Launching Ceremony. Geneva: World Health Organization (2021).

[12] WHO. COVID-19 Advice for the Public: Getting Vaccinated. Geneva: World Health Organization (2021).

[13] Russell FM Greenwood B. Who should be prioritised for COVID-19 vaccination? Hum Vacc Immunother. (2021) 17:1317–21. 10.1080/21645515.2020.1827882 33141000 PMC8078651

[14] Halim M Halim A Tjhin Y. COVID-19 vaccination efficacy and safety literature review. J Clin Med Res. (2021) 3:1–10. 10.37191/Mapsci-2582-4333-3(1)-058

[15] Dubé E Laberge C Guay M Bramadat P Roy R Bettinger JA. Vaccine hesitancy: an overview. Hum Vacc Immunother. (2013) 9:1763–73. 10.4161/hv.24657 23584253 PMC3906279

[16] Lazarus JV Ratzan SC Palayew A Gostin LO Larson HJ Rabin K. A global survey of potential acceptance of a COVID-19 vaccine. Nat Med. (2021) 27:225–8. 10.1038/s41591-020-1124-9 33082575 PMC7573523

[17] Voysey M Clemens SAC Madhi SA Weckx LY Folegatti PM Aley PK. Safety and efficacy of the ChAdOx1 nCoV-19 vaccine (AZD1222) against SARS-CoV-2: an interim analysis of four randomised controlled trials in Brazil, South Africa, and the UK. Lancet. (2021) 397:99–111. 10.1016/S0140-6736(20)32661-1 33306989 PMC7723445

[18] Agyekum MW Afrifa-Anane GF Kyei-Arthur F Addo B. Acceptability of COVID-19 vaccination among health care workers in Ghana. Adv Public Health. (2021) 2021:9998176. 10.1101/2021.03.11.21253374

[19] Kasozi KI Laudisoit A Osuwat LO Batiha GE-S Al Omairi NE Aigbogun E. A descriptive-multivariate analysis of community knowledge, confidence, and trust in COVID-19 clinical trials among healthcare workers in Uganda. Vaccines. (2021) 9:253. 10.3390/vaccines9030253 33809269 PMC8000597

[20] Nzaji MK Ngombe LK Mwamba GN Ndala DBB Miema JM Lungoyo CL. Acceptability of vaccination against COVID-19 among healthcare workers in the democratic Republic of the Congo. Pragm Observ Res. (2020) 11:103. 10.2147/POR.S271096 33154695 PMC7605960

[21] Mwai P. COVID-19 Africa: What is Happening with Vaccines? London: BBC (2021).

[22] Africa C. Majority of Africans would take a safe and effective COVID-19 vaccine. Addis Ababa: African Union (2021), 12.

[23] WHO. COVID-19-Vaccine Country Readiness and Delivery/Acceptance and Demand. Geneva: WHO (2021).

[24] Zhang K Fang Y. Chan PS-f Cao H Chen H Hu T. Behavioral intention to get a booster dose of COVID-19 vaccine among Chinese factory workers. Int J Environ Res Public Health. (2022) 19:5245. 10.3390/ijerph19095245 35564639 PMC9099970

[25] Adane M Ademas A Kloos H. Knowledge, attitudes, and perceptions of COVID-19 vaccine and refusal to receive COVID-19 vaccine among healthcare workers in northeastern Ethiopia. BMC Public Health. (2022) 22:1–14. 10.1186/s12889-021-12362-8 35042476 PMC8765812

[26] Al-Zalfawi SM Rabbani SI Asdaq SMB Alamri AS Alsanie WF Alhomrani M. Public Knowledge, attitude, and perception towards COVID-19 vaccination in Saudi Arabia. Int J Environ Res Public Health. (2021) 18:10081. 10.3390/ijerph181910081 34639382 PMC8508088

[27] Li H Cheng L Tao J Chen D Zeng C. Knowledge and willingness to receive a COVID-19 vaccine: a survey from Anhui Province, China. Hum Vacc Immunother. (2022) 2022:1–8. 10.1080/21645515.2021.2024064 35130110 PMC8993089

[28] Brüssow H. COVID-19: vaccination problems. Environ Microbiol. (2021) 23:2878–90. 10.1111/1462-2920.15549 33928745 PMC8209888

[29] Al-Qerem WA Jarab AS. COVID-19 vaccination acceptance and its associated factors among a middle eastern population. Front Public Health. (2021) 9:632914. 10.3389/fpubh.2021.632914 33643995 PMC7902782

[30] Wang Q Yang L Jin H Lin L. Vaccination against COVID-19: a systematic review and meta-analysis of acceptability and its predictors. Prev Med. (2021) 150:106694. 10.1016/j.ypmed.2021.106694 34171345 PMC8217737

[31] Kourlaba G Kourkouni E Maistreli S Tsopela C-G Molocha N-M Triantafyllou C. Willingness of Greek general population to get a COVID-19 vaccine. Global Health Res Policy. (2021) 6:1–10. 10.1186/s41256-021-00188-1 33509291 PMC7843240

[32] Bhartiya S Kumar N Singh T Murugan S Rajavel S Wadhwani M. Knowledge, attitude and practice towards COVID-19 vaccination acceptance in West India. Int J Commun Med Public Health. (2021) 8:1170–6. 10.18203/2394-6040.ijcmph20210481

[33] Kumari A Ranjan P Chopra S Kaur D Kaur T Upadhyay AD. Knowledge, barriers and facilitators regarding COVID-19 vaccine and vaccination programme among the general population: a cross-sectional survey from one thousand two hundred and forty-nine participants. Diabet Metabol Synd Clin Res Rev. (2021) 15:987–92. 10.1016/j.dsx.2021.04.015 33984818 PMC8087578

[34] Wong MC Wong EL Huang J Cheung AW Law K Chong MK. Acceptance of the COVID-19 vaccine based on the health belief model: a population-based survey in Hong Kong. Vaccine. (2021) 39:1148–56. 10.1016/j.vaccine.2020.12.083 33461834 PMC7832076

[35] Abedin M Islam MA Rahman FN Reza HM Hossain MZ Hossain MA. Willingness to vaccinate against COVID-19 among Bangladeshi adults: understanding the strategies to optimize vaccination coverage. PLoS ONE. (2021) 16:e0250495. 10.1371/journal.pone.0250495 33905442 PMC8078802

[36] Alfageeh EI Alshareef N Angawi K Alhazmi F Chirwa GC. Acceptability of a COVID-19 vaccine among the Saudi population. Vaccines. (2021) 9:226. 10.3390/vaccines9030226 33807732 PMC7999879

[37] Mahmud S Mohsin M Khan IA Mian AU Zaman MA. Acceptance of COVID-19 vaccine and its determinants in Bangladesh. arXiv preprint arXiv:210315206 (2021). 10.1371/journal.pone.0257096 PMC8428569 34499673

[38] Yanto TA Octavius GS Heriyanto RS Ienawi C Nisa H Pasai HE. Psychological factors affecting COVID-19 vaccine acceptance in Indonesia. Egy J Neurol Psychiatry Neurosurg. (2021) 57:1–8. 10.1186/s41983-021-00436-8 34955630 PMC8685827

[39] Barbuscia A Comolli C. Gender and socioeconomic inequalities in health and wellbeing across age in France and Switzerland. Vienna Yearbook Populat Res. (2021) 19:TBA-OLF. 10.1553/populationyearbook2021.res2.232672529

[40] Zakar R Momina AU Shahzad S Hayee M Shahzad R Zakar MZ. COVID-19 vaccination hesitancy or acceptancea and its associated factors: findings from post-vaccination cross-sectional survey from Punjab Pakistan. Int J Environ Res Public Health. (2022) 19:1305. 10.3390/ijerph19031305 35162328 PMC8835289

[41] Lai X Zhu H Wang J Huang Y Jing R Lyu Y. Public perceptions and acceptance of COVID-19 booster vaccination in China: a cross-sectional study. Vaccines. (2021) 9:1461. 10.3390/vaccines9121461 34960208 PMC8709447

[42] Machida M Nakamura I Kojima T Saito R Nakaya T Hanibuchi T. Acceptance of a COVID-19 vaccine in Japan during the COVID-19 pandemic. Vaccines. (2021) 9:210. 10.3390/vaccines9030210 33802285 PMC8002097

[43] Shmueli L. Predicting intention to receive COVID-19 vaccine among the general population using the health belief model and the theory of planned behavior model. BMC Public Health. (2021) 21:804. 10.1186/s12889-021-10816-7 33902501 PMC8075011

[44] Raingruber B. Health Promotion Theories. Contemporary Health Promotion in Nursing Practice. Burlington: Jones & Bartlett Publishers (2014).

[45] Wismans A Thurik R Baptista R Dejardin M Janssen F Franken I. Psychological characteristics and the mediating role of the 5C Model in explaining students' COVID-19 vaccination intention. PLoS ONE. (2021) 16:e0255382. 10.1371/journal.pone.0255382 34379648 PMC8357093

[46] Al-Metwali BZ Al-Jumaili AA Al-Alag ZA Sorofman B. Exploring the acceptance of COVID-19 vaccine among healthcare workers and general population using health belief model. J Eval Clin Pract. (2021) 27:1112–22. 10.1111/jep.13581 33960582 PMC8242385

